# Conditional Inactivation of *Brca1*, *p53* and *Rb* in Mouse Ovaries Results in the Development of Leiomyosarcomas

**DOI:** 10.1371/journal.pone.0008534

**Published:** 2009-12-31

**Authors:** Katherine V. Clark-Knowles, Mary K. Senterman, Olga Collins, Barbara C. Vanderhyden

**Affiliations:** 1 Department of Cellular and Molecular Medicine, University of Ottawa, Ottawa, Ontario, Canada; 2 Department of Pathology and Laboratory Medicine, University of Ottawa, Ottawa, Ontario, Canada; 3 Department of Obstetrics and Gynecology, University of Ottawa, Ottawa, Ontario, Canada; 4 Ottawa Hospital Research Institute, Ottawa, Ontario, Canada; Cincinnati Children's Research Foundation, United States of America

## Abstract

Epithelial ovarian cancer (EOC) is thought to arise in part from the ovarian surface epithelium (OSE); however, the molecular events underlying this transformation are poorly understood. Germline mutations in the *BRCA1* tumor suppressor gene result in a significantly increased risk of developing EOC and a large proportion of sporadic EOCs display some sort of *BRCA1* dysfunction. To generate a model in which *Brca1*-mediated transformation can be studied, we previously inactivated *Brca1* alone in murine OSE, which resulted in an increased accumulation of premalignant changes, but no tumor formation. In this study, we examined tumor formation in mice with conditionally expressed alleles of *Brca1*, *p53* and *Rb*, alone or in combination. Intrabursal injection of adenovirus expressing Cre recombinase to inactivate *p53* resulted in tumors in 100% of mice. Tumor progression was accelerated in mice with concomitant inactivation of *Brca1* and *p53*, but not *Rb* and *p53*. Immunohistologic analyses classified the tumors as leiomyosarcomas that may be arising from the ovarian bursa. *Brca1* inactivation in primary cultures of murine OSE cells led to a suppression of proliferation that could be rescued by concomitant inactivation of *p53* and/or *Rb*. *Brca1*-deficient OSE cells displayed an increased sensitivity to the DNA damaging agent cisplatin, and this effect could be modulated by inactivation of *p53* and/or *Rb*. These results indicate that *Brca1* deficiency can accelerate tumor development and alter the sensitivity of OSE cells to chemotherapeutic agents. Intrabursal delivery of adenovirus intended to alter gene expression in the ovarian surface epithelium may, in some strains of mice, result in more rapid transformation of adjacent cells, resulting in leiomyosarcomas.

## Introduction

Epithelial ovarian cancer (EOC) is thought to arise from the ovarian surface epithelium (OSE) [1], although recent evidence implicates the fallopian tube as a potential tissue of origin of EOC of the serous histotype [2]–[5]. Embryonically derived from the coelemic epithelium, the OSE is a single layer of squamous to cuboidal cells that demonstrates a plastic phenotype reflecting its ability to undergo epithelio-mesenchymal transition [6]. With age and repeated ovulatory cycles, the OSE assumes a more irregular contour and forms invaginations or clefts into the stroma, which may pinch off completely and form epithelial inclusion cysts within the ovary. These crypts and cysts often show evidence of early metaplastic changes in cell shape and express markers up-regulated in ovarian tumors, such as E-cadherin [7] and CA125 [1], which suggests that these premalignant lesions may give rise to ovarian neoplasias. Prophylactic oophorectomy specimens from women at high risk for developing ovarian cancer, due to a strong family history of the disease or the presence of a germline mutation in *BRCA1*, have more of these morphological changes in the OSE than ovaries removed incidentally during total abdominal hysterectomy [8]–[10]. In one study, 6% of prophylactically removed ovaries from *BRCA1* mutation carriers were found to harbor microscopic ovarian carcinomas [11].

Five to fifteen percent of ovarian cancers are thought to be due to hereditary factors and the majority of these can be attributed to germline mutations in the *BRCA1* gene [12], [13]. These germline mutations confer a lifetime risk of ovarian cancer up to 60% compared to ∼2% in the general population [14], [15]. Although somatic mutations in *BRCA1* are rare, reduced or absent protein expression has been observed in up to 90% of sporadic ovarian tumors indicating that epigenetic factors, mainly promoter hypermethylation, are also involved in its regulation [16]–[18]. The *BRCA1* gene has been implicated in a wide variety of cellular processes, including maintenance of genome integrity [19], DNA damage recognition and repair [20], [21], cell cycle checkpoint control [22], [23], and apoptosis [24].

Up to 60% of *BRCA1* mutation-associated ovarian tumors also display mutations in the *p53* tumor suppressor gene [25], [26]. Mouse models of mammary tumorigenesis have revealed a role for p53 in Brca1-related transformation [27]–[29]. In mice in which *Brca1* was inactivated in the mammary epithelium, the latency of tumor formation could be shortened by the concomitant inactivation of the *p53* tumor suppressor gene [30], [31]. Downregulaton of BRCA1 results in increased p53 and p21 expression [31], which may represent a significant obstacle to tumorigenesis that can be overcome by somatic mutation of the p53 gene.

The ability of BRCA1 to suppress cellular proliferation may depend, at least in part, on its association with the retinoblastoma tumor suppressor (RB), since RB preferentially binds to exon 11 of *BRCA1* [32]. RB has also been shown to modify *BRCA1* expression via its modulation of *E2F* transcriptional activity, with *BRCA1* being an *in vivo* target of *E2F1* [33]. And overexpression of *BRCA1* inhibits the expression of *RB* and the *RB* family members *p107* and *p130* [34]. No association has yet been made between *RB* and *BRCA1* dysfunction in ovarian cancer.

The *Brca1*, *Rb* and *p53* tumor suppressors have been conditionally inactivated in mouse OSE to study their roles in ovarian epithelial cell transformation. Simultaneous inactivation of *p53* and *Rb* in the mouse OSE led to the development of malignant ovarian tumors [35]. We inactivated *Brca1* in the murine OSE which resulted in the increased accumulation of premalignant changes, although no tumor formation was observed after one year [36]. However, inactivation of *Brca1* in cultured murine OSE (MOSE) cells resulted in a slowed proliferation that could be rescued by concurrent inactivation of *p53* [36]. In the present study, a conditional and site-directed strategy was employed to test whether inactivation of *Brca1, p53*, and *Rb* in various combinations in the OSE is sufficient to establish a mouse model of Brca1-associated ovarian cancer. The ovaries of these mice were examined at various time points to determine if there were progressive alterations in epithelial morphology or tumor formation as has been observed in human prophylactic oophorectomy specimens. Primary cultures of OSE cells from these mice were also established to determine the impact of combined inactivation of these genes on their rate of proliferation and sensitivity to cisplatin.

## Materials and Methods

### Experimental Animals

*Brca1* ^loxP/loxP^ [FVB;129-*Brca1^tm2Brn^*] conditional knockout mice, bearing loxP sites in introns 4 and 13 of the *Brca1* gene, and *p53* ^loxP/loxP^ [FVB;129-*Trp53^tm1Brn^*] [37] mice bearing loxP in introns 1 and 10 of the *p53* gene were obtained from the Mouse Models of Human Cancers Consortium Mouse Repository (National Cancer Institute, Rockville, MD, USA). Rb^loxP/loxP^ mice [129sv-*Rb1* ^tm2Brn^] [38], bearing loxP sites flanking exon 19 of the *Rb* gene, were obtained from Dr. Ruth Slack at the University of Ottawa (Ottawa, Ontario, Canada). The mice were intercrossed through multiple generations to create colonies of homozygous *Brca1* ^loxP/loxP^/*p53* ^loxP/loxP^, *Rb^l^* ^oxP/loxP^/*p53* ^loxP/loxP^, and *Rb* ^loxP/loxP^/*Brca1* ^loxP/loxP^ double conditional knockout mice. These mice were then intercrossed through successive generations in order to obtain *Brca1* ^loxP/loxP^/*Rb* ^loxP/loxP^/*p53* ^loxP/loxP^ triple conditional knockout mice. All animal experiments described in this study were performed according to the *Guidelines for the Care and Use of Animals* established by the Canadian Council on Animal Care. Recombinant adenoviruses Ad5CMVeGFP (AdGFP) or Ad5CMVCre (AdCre) (Vector Development Laboratory, Houston, TX) were delivered to the OSE *in vivo* via intrabursal injection as previously described [36].

### Genotyping and Detection of Recombination

Genomic DNA was extracted from ear punches or cultured cells as previously described [36]. *Brca1* ^loxP/loxP^ mice were genotyped as previously described [39]. *p53* ^loxP/loxP^ mice were genotyped using primers p53int1fwd (5′ CAC AAA AAC AGG TTA AAC CAG 3′) and p53int1rev (5′ AGC ACA TAG GAG GCA GAG AC 3′) to yield a 288 bp band for wild-type or a 370 bp band for floxed sequences or primers p53int10fwd (5′ AAG GGG TAT GAG GGA CAA GG 3′) and p53int10rev (5′ GAA GAC AGA AAA GGG GAG GG 3′), which yield a 431 bp or a 584 bp fragment for wild-type and floxed sequences respectively. For detection of Cre-mediated excision of exons 2–10 of *p53* (hereafter designated *p53* ^Δ2-10^), primers p53int1fwd and p53int10rev were used, yielding a 612 bp product. Remaining unrecombined sequence following Cre exposure was detected using primers p53int1fwd and p53int1rev or primers p53int10fwd and p53int10rev in a separate amplification. *Rb* ^loxP/loxP^ mice were genotyped using primers Rb18 (5′ GGC GTG TGC CAT CAA TG 3′) and Rb19 (5′ AAC TCA AGG GAG ACC TG 3′). DNA from wild-type mice yielded a 750 bp band, while floxed mice were identified by the presence of a 650 bp band. For detection of Cre-mediated excision of exon 19 of the *Rb* gene (hereafter designated *Rb* ^Δ19^), primers Rb18 and Rb212 (5′ GAA AGG AAA GTC AGG GAC ATT GGG 3′) were used, yielding a 260 bp product when recombination has occurred and a 750 bp product for the unrecombined fraction.

### Tissue Collection

Animals were euthanized by CO_2_ asphyxiation at 60, 120, 180, or 240 days post-injection of AdGFP or AdCre. The ovaries (with the ovarian fat pad and bursa intact) were removed individually along with the attached oviduct and a portion of the uterus, fixed in formalin and paraffin-embedded. Five µm serial sections were cut for H&E staining and morphological changes to the OSE were assessed by examining five non-consecutive sections of each ovary at 200× magnification using an Olympus BX50 microscope (Olympus, Melville, NY, USA). Samples were blinded prior to examination. Sections were evaluated for the number of areas of columnar cells, areas of hyperplasia, and invaginations of the OSE, as described previously [36]. Mice that developed tumors were euthanized when they had reached a loss-of-wellness endpoint due to tumor burden, defined as any or all of the following: presence of respiratory distress, rapid weight loss or gain >5 g from the average body weight of control mice of the same age, and presence of a palpable mass or abdominal distention that impairs mobility. Tumors were removed, fixed in formalin and paraffin embedded, with a small piece of tissue reserved for genomic DNA isolation. Serial sections of tumor tissue with a thickness of 5 µm were cut for H&E staining and immunohistochemistry.

### Immunohistochemistry

Paraffin sections were deparaffinized in xylene and rehydrated in ethanol according to standard protocol. High temperature antigen retrieval was performed using sodium citrate buffer (pH 6.0) and endogenous peroxidase activity was blocked using 3% hydrogen peroxide in Stockholm PBS (S-PBS). All samples were blocked using an avidin/biotin blocking kit (DAKO, Cytomation, Carpentaria, CA, USA). Primary antibodies were diluted in S-PBS at the following concentrations: rat anti-CK19, 1∶100 (TROMA-1, Developmental Studies Hybridoma Bank, University of Iowa, USA); rat anti-Ki67, 1∶25 (DAKO); mouse-anti smooth muscle actin (SMA), 1∶100 (DAKO); mouse anti-Desmin, 1∶100 (DAKO); and rat anti-CD34, 1∶50 (Novus Biologicals). For antibodies raised in rat, sections were incubated with primary antibody at room temperature for 2 hours or overnight. Following three 5 min washes in S-PBS, sections were stained with an anti-rabbit secondary antibody (1∶200, DAKO) for 20 min followed by three 5 min washes and incubation with a streptavidin/horseradish peroxidase solution (1∶200, DAKO) for 20 min. For antibodies raised in mice, the Vector® Mouse on Mouse™ immunodetection kit (Vector Laboratories, Burlingame, CA, USA) was used according to manufacturer specifications. Developing was performed with diaminobenzidine (DAB) as a substrate (0.2% DAB, 0.001% H_2_O_2_; Sigma-Aldrich). Slides were counterstained with hematoxylin, dehydrated, and coverslipped with Permount mounting medium (Fisher Scientific, Ottawa, ON, Canada).

### MOSE Proliferation and Sensitivity to Cisplatin

Primary cultures of MOSE cells were established and maintained as previously described [36]. For *in vitro* infections, 5.0×10^5^ MOSE cells of the various genotypes were infected with either AdCre or AdGFP following an established protocol [35], and experiments to evaluate proliferation and cisplatin sensitivity were performed following cell replating 72 hours later, as described previously [36]. Proliferation of MOSE cells was also assessed several passages (4–9) after infection. Experiments were performed three times in triplicate, with a separate infection for each replicate.

### Statistical Analyses

Cell counts are expressed as the mean±SEM of three independent experiments performed in triplicate. The probability of significant differences was determined by Student's *t* test (two groups) and by analysis of variance (ANOVA; multiple groups). Bonferroni's post-test was used to determine significance between specific treatments when whole group differences were detected by ANOVA. Survival curves were compared using a Log-Rank test. For all analyses, significance was inferred at P<0.05 and P values were two-sided. Analyses were performed using Graphpad Prism statistical software (Graphpad Software, San Diego, CA, USA).

## Results

### Conditional Inactivation of *Brca1* in Conjunction with *p53* and/or *Rb* Using Intrabursal Injection

We have previously shown that inactivation of *Brca1* alone in the OSE resulted in an increase in the number of preneoplastic changes in the OSE after 240 days, but no tumor formation [36]. In this study we observed that no tumors arose in mice in which *Rb* alone was conditionally inactivated, even after one year following intrabursal injection of AdCre. There were no significant differences in the number of morphological changes in the OSE between the AdCre-treated *Rb* ^Δ19^ mice and the control *Rb* ^loxP/loxP^ mice at any of the time points examined (**Table 1**). There was no significant age-related increase in the total number of changes, as was previously observed in the *Brca1* ^Δ5-13^ mice. However, the mean number of morphological changes in the OSE doubled between 120 and 180 days in the *Rb* ^Δ19^ mice (4.32±0.78 versus 8.52±0.80 mean changes per section over five non-consecutive sections per ovary, P<0.05), whereas this difference was not noted in the *Rb* ^loxP/loxP^ mice.

**Table 1:** Distribution of morphological features in the OSE over time following conditional inactivation of *Rb*.

	Time (Days)			
Epithelial Morphology	60	120	180	240
**Areas of Columnar Cells**				
*** Rb*** **^Δ19^**	3.34±0.33^a^ (n = 10)	2.38±0.49^a^ (n = 10)	4.00±0.53^a^ (n = 10)	4.33±0.67^a^ (n = 8)
*** Rb*** **^loxP/loxP^**	2.48±0.28^a^ (n = 10)	2.99±0.53^a^ (n = 8)	4.76±0.53^b^ (n = 10)	4.02±0.36^a^ (n = 10)
**Areas of Hyperplasia**				
*** Rb*** **^Δ19^**	2.60±0.77^a^	1.90±0.54^a^	4.52±0.67^a^	2.08±0.38^a^
*** Rb*** **^loxP/loxP^**	2.43±0.69^a^	2.96±0.93^a^	3.04±0.70^a^	2.32±0.50^a^
**Epithelial Invaginations**				
*** Rb*** **^Δ19^**	0.13±0.07^a^	0.04±0.04^a^	0^a^	0.06±0.06^a^
*** Rb*** **^loxP/loxP^**	0^a^	0.03±0.03^a^	0^a^	0^a^
**Total Changes**				
*** Rb*** **Δ^19^**	6.05±0.86^a^	4.32±0.78^a^	8.52±0.80^b^	6.40±0.80^a^
*** Rb*** **^loxP/loxP^**	4.90±0.79^a^	5.98±1.16^a^	7.80±1.10^a^	6.40±0.63^a^

Numbers represent the mean±SEM number of morphological changes per section over five non-consecutive sections in n ovaries. Superscript letters denote a significant difference between time points for that treatment group.

Tumor formation was also not observed in mice in which both *Rb* and *Brca1* were concomitantly inactivated via intrabursal injection of AdCre. The number of morphological changes in the OSE increased over time in both groups, however there were no significant differences between the *Rb* ^Δ19^/*Brca1* ^Δ5-13^ ovaries and the *Rb* ^loxP/loxP^/*Brca1* ^loxP/loxP^ ovaries at any time point examined (**Table 2**). There was a significant increase in the number of areas of columnar cells between 60 and 240 days in the *Rb* ^Δ19^/*Brca1* ^Δ5-13^ mice (1.96±0.45 versus 5.18±0.70, P<0.01) that was not seen in the *Rb^l^* ^oxP/loxP^/*Brca1* ^loxP/loxP^ mice. As well, epithelial invaginations were noted only in the OSE of the *Rb* ^Δ19^/*Brca1* ^Δ5-13^ mice.

**Table 2:** Distribution of morphological features in the OSE over time following conditional inactivation of *Rb* and *Brca1*.

	Time (Days)			
Epithelial Morphology	60	120	180	240
**Areas of Columnar Cells**				
*** Rb*** **^Δ19^/** ***Brca1*** **^Δ5-13^**	1.96±0.45^a^ (n = 10)	4.00±0.59^a^ (n = 10)	4.27±0.49^a^ (n = 12)	5.18±0.70^b^ (n = 10)
*** Rb*** **^loxP/loxP^/** ***Brca1*** **^loxP/loxP^**	3.24±0.43^a^ (n = 10)	3.38±0.31^a^ (n = 10)	3.30±0.32^a^ (n = 12)	5.02±0.85^a^ (n = 10)
**Areas of Hyperplasia**				
*** Rb*** **^Δ19^/** ***Brca1*** **^Δ5-13^**	2.64±0.68^a^	2.70±0.84^a^	4.23±0.75^a^	4.14±0.84^a^
*** Rb*** **^loxP/loxP^/** ***Brca1*** **^loxP/loxP^**	1.76±0.40^a^	2.80±0.62^a^	3.08±0.68^a^	2.42±0.87^a^
**Epithelial Invaginations**				
*** Rb*** **^Δ19^/** ***Brca1*** **^Δ5-13^**	0	0	0.35±0.25	0.20±0.11
*** Rb*** **^loxP/loxP^/** ***Brca1*** **^loxP/loxP^**	0	0	0	0
**Total Changes**				
*** Rb*** **^Δ19^/** ***Brca1*** **^Δ5-13^**	4.62±1.08^a^	8.85±1.06^a^	8.52±0.80^a^	9.52±1.51^a^
*** Rb*** **^loxP/loxP^/** ***Brca1*** **^loxP/loxP^**	5.00±0.72^a^	6.38±0.90^a^	7.80±1.10^a^	7.44±1.62^a^

Numbers represent the mean±SEM of the number of morphological changes per section over five non-consecutive sections in n ovaries. Letters are used to denote a significant difference between time points for that treatment group.

**Figure 1:**
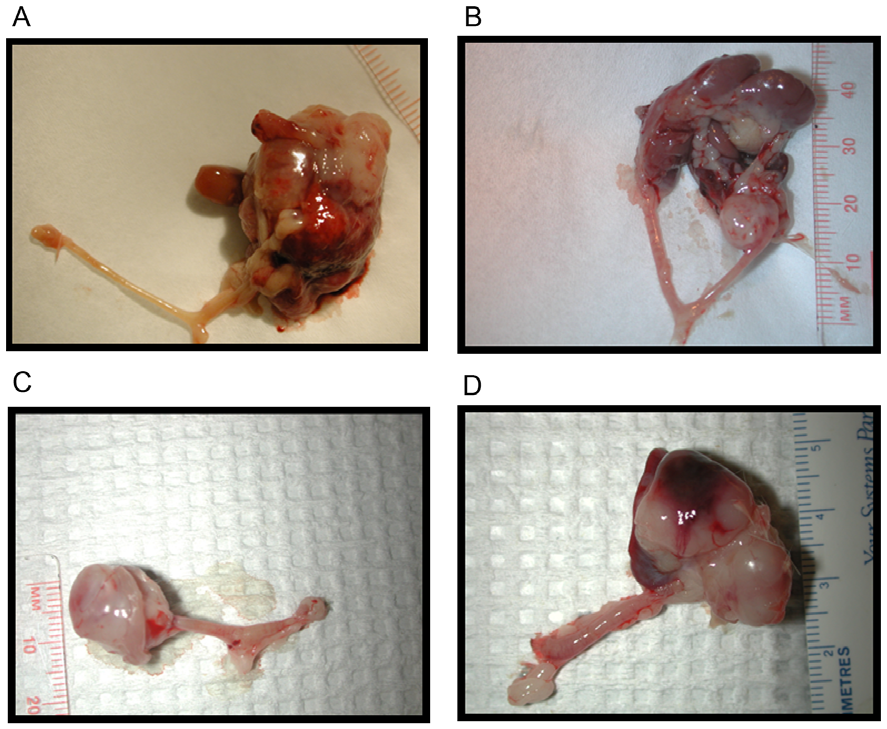
Gross tumor presentation of mice at loss-of-wellness endpoint following intrabursal administration of AdCre. Representative images of tumors arising in mice in which *Brca1*, *p53*, and/or *Rb* had been inactivated in the OSE: *p53* ^Δ2-10^ (**A**), *Brca1* ^Δ5-13^/*p53* ^Δ2-10^ (**B**), *Rb* ^Δ19^/*p53* ^Δ2-10^ (**C**), and *Rb* ^Δ19^/*p53* ^Δ2-10^/*Brca1* ^Δ5-13^ (**D**).

**Figure 2:**
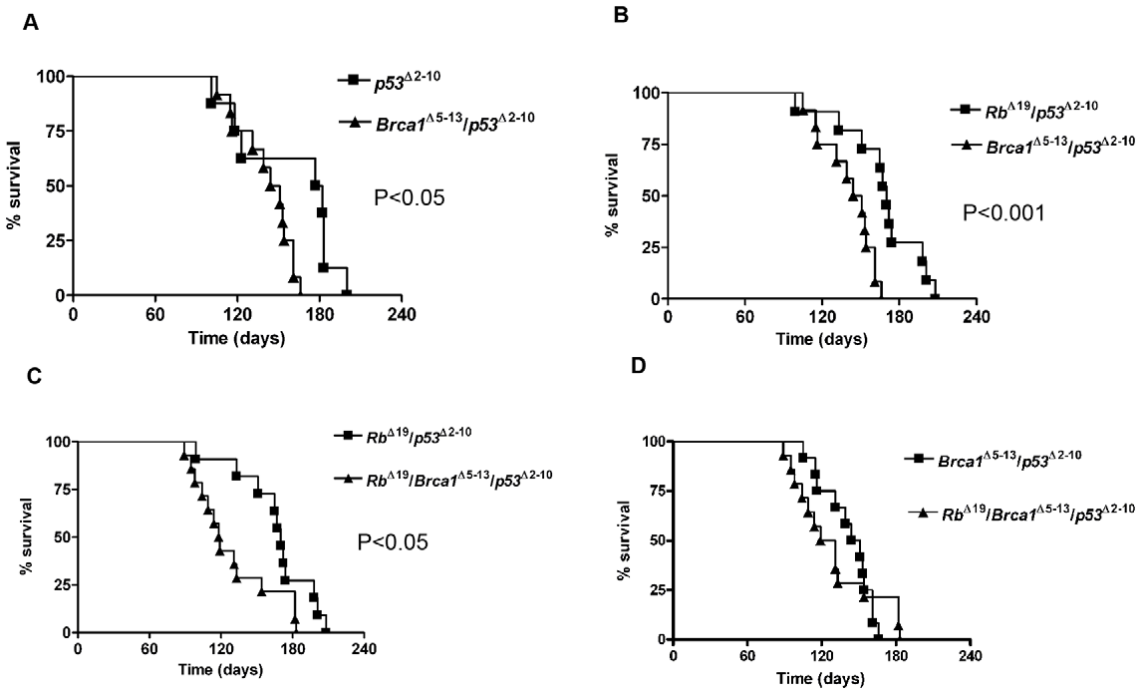
Survival of mice following inactivation of multiple tumor suppressors in the OSE *in vivo*. Kaplan-Meier plots showing the time to loss-of-wellness endpoint of the mice in which *Brca1*, *p53*, and/or *Rb* had been inactivated in the OSE. **A**) The median survival time of the *Brca1* ^Δ5-13^/*p53* ^Δ2-10^ mice (147.5 days) is significantly shorter than that of the *p53* ^Δ2-10^ mice (179.5 days), P<0.05. **B**) The median survival time of the *Brca1* ^Δ5-13^/*p53* ^Δ2-10^ mice (147.5 days) is significantly less than that of the *Rb* ^Δ19^/*p53* ^Δ2-10^ mice (170 days), P<0.001. **C**) The median survival time of the *Rb* ^Δ19^/*p53* ^Δ2-10^/*Brca1* ^Δ5-13^ mice (118.5 days) is significantly less than that of the *Rb* ^Δ19^/*p53* ^Δ2-10^ mice (170 days), P<0.05. **D**) The median survival time of the *Rb* ^Δ19^/*p53* ^Δ2-10^/*Brca1* ^Δ5-13^ mice (118.5 days) is less than that of the *Brca1* ^Δ5-13^/*p53* ^Δ2-10^ mice (147.5 days), although the difference in survival was not statistically significant.

Tumor formation was observed following intrabursal injection of AdCre to generate cells with the following genotypes: *p53* ^Δ2-10^, *Rb* ^Δ19^/*p53* ^Δ2-10^, *Brca1* ^Δ5-13^/*p53* ^Δ2-10^, and *Rb* ^Δ19^/*p53* ^Δ2-10^/*Brca1* ^Δ5-13^ (**Table 3**). No tumors were observed in any of the mice injected with adenoviral GFP when followed to one year post-intrabursal injection. The presentation of the mice at endpoint was similar in all of the groups examined, with either a large, palpable dorsal mass(es) or severe abdominal distention. The period between onset of symptoms and loss-of-wellness endpoint was quite short, generally one week or less. **Figure 1** illustrates the general appearance of the tumors upon necropsy for each genotype. The tumors were most often located at the end of the uterine horn involving the ovary and sometimes engulfing surrounding organs such as the pancreas and the spleen. Both ovaries were subjected to intrabursal injection in all animals, however bilateral tumors were only observed in the *Rb* ^Δ19^/*p53* ^Δ2-10^/*Brca1* ^Δ5-13^ mice, and only in 2 of 14 animals. The incidence, median survival, range of survival and presence of ascites for these groups of mice are summarized in **Table 3** with the Kaplan-Meier survival plots depicted in **Figure 2**. When compiled, these data indicate that loss of *p53* is necessary for tumor formation and that *Brca1* deficiency accelerates tumor initiation and/or progression.

**Table 3:** Tumor formation following intrabursal injection of adenoviral Cre recombinase.

Genotype	# of mice^a^	# of mice with tumors	Median survival in days (range)	% with ascites
*Brca1* ^Δ5-13^	5	0		
*Rb* ^Δ19±^	5	0		
*Rb* ^Δ19^/*Brca1* ^Δ5-13^	5	0		
*p53* ^Δ2-10^	8	8	179.5 (101–200)	50
*Rb* ^Δ19^/*p53* ^Δ2-10^	10	10	170 (99–207)	30
*Brca1* ^Δ5-13^/*p53* ^Δ2-10^	12	12	147.5 (105–166)	42
*Rb* ^Δ19^/*p53* ^Δ2-10^/*Brca1* ^Δ5-13^	14	14	118.5 (89–183)	0

a Number of mice indicates the number of animals who received intrabursal injection of adenoviral Cre recombinase and were euthanized when they had reached a loss-of-wellness endpoint due to tumor burden or had reached the 240 day time point.

**Figure 3:**
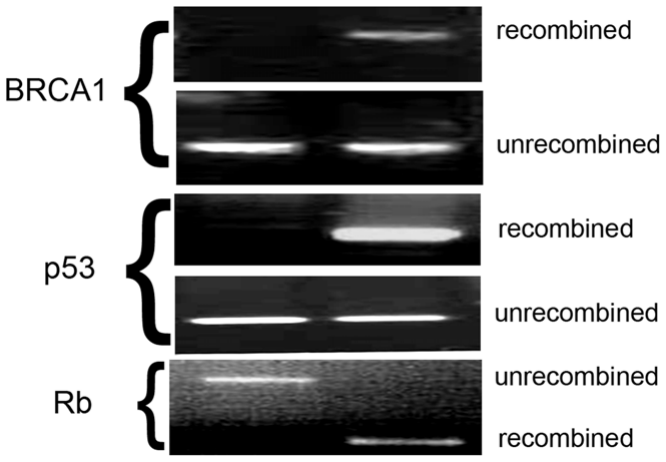
Recombination at loxP sites in the relevant tumor suppressor genes in tumor samples. PCR of genomic DNA extracted from the tumor and the corresponding tail (control for lack of recombination) to detect recombination at loxP sites of the tumor suppressor genes relevant to the genotype of the mouse.

Recombination at the relevant loxP sites was confirmed in genomic DNA from all the tumor samples (**Figure 3**). Unrecombined DNA was also detectable in all tumor samples, which was not unexpected given that the tumors are likely composed of both tumor cells and the cells of the normal tissue they have invaded. Recombination at the relevant loxP sites was also detectable in genomic DNA from ascites cells isolated from tumor-bearing mice (data not shown).

### Histopathologic Features of Tumors in Mice with Conditional Inactivation of *Brca1*, *p53* and *Rb*

**Figure 4:**
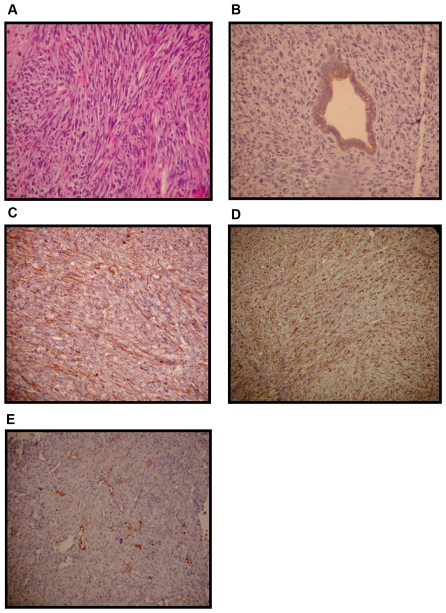
Representative images of tumor histology. Paraffin sections (5 µm) of the tumors were stained with H&E or specific antibodies to examine histology and gene expression: **A**) H&E (200X) **B**) cytokeratin19 (400X) **C**) smooth muscle actin (200X) **D**) desmin (100X) **E**) CD34 (100X). Antigen detection in B–E is by the presence of a brownish-red stain.

There was no obvious difference in the pathology of the tumors as related to their genotype. H&E stained sections revealed densely-packed and highly malignant cells with spindle-shape morphology, as well as the presence of anaplastic giant cells in all of the samples examined (**Figure 4A**). Further immunohistochemical analyses revealed that the tumors were predominantly negative for the epithelial marker cytokeratin (CK) 19, with the exception of some glandular structures present in a small number of the tumors (**Figure 4B**). These tumors were also probed for the presence of other epithelial markers such as CK8 and pan-cytokeratin and were found to be negative with the exception of the areas noted above. All of the tumors examined were positive for smooth muscle actin (SMA) (**Figure 4C**) and desmin (**Figure 4D**), both smooth muscle markers, and were negative for CD34, a hematological marker (**Figure 4E**). Pathologic review of tumor histology and immunohistochemical data indicates that these tumors are malignant leiomyosarcomas.

**Figure 5:**
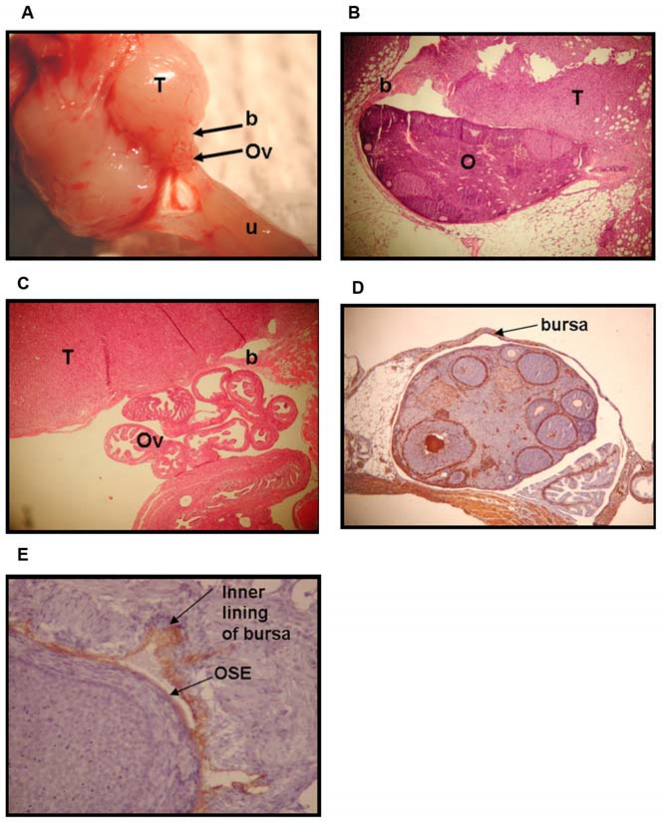
Tumors appear to be associated with the ovarian bursal membrane. Gross anatomical image (**A**) and H&E sections (**B** and **C**) showing involvement of ovarian bursal membrane with tumor. T = tumor, b = bursal membrane, Ov = oviduct, O = ovary, and u = uterus (25X). **D**) SMA staining (reddish-brown stain) of a normal ovary with an intact bursal membrane (25X) **E**) CK19 staining (reddish-brown stain) of a normal ovary with an intact bursal membrane (400X).

The gross pathology along with the H&E sections provided some clues as to the origin of the tumors. In some instances, normal ovary was visible at the edge of a tumor, such that the bursal membrane that surrounds the ovary was flush with the tumor (**Figure 5A**). In some cases, even when normal ovary was not grossly visible at necropsy, the ovary was found within the H&E sections of the tumor (**Figure 5B**). In these sections, the tumors also appeared to be connected to the bursal membrane (**Figure 5C**). Staining of sections of normal ovaries revealed that the bursa, which is an extension of the oviduct, is highly positive for SMA (**Figure 5D**). Staining also revealed that the inner lining of the bursal membrane is positive for CK19 (**Figure 5E**). Ovaries from the *p53* ^Δ2-10^, *Rb* ^Δ19^/*p53* ^Δ2-10^, Brca1^Δ5-13^/p53^Δ2-10^, and *Rb* ^Δ19^/*Brca1* ^Δ5-13^/*p53* ^Δ2-10^ mice at early time points (60 and 120 days) were uninformative in terms of potential precursor lesions for these tumors. There were no advanced morphological changes (invaginations or inclusion cysts) noted in ovaries collected from the mice at 60 or 120 days post-AdCre injection (data not shown).

### Effect of Concomitant Inactivation of Multiple Tumor Suppressor Genes on Proliferation of MOSE Cells *In Vitro*

**Figure 6:**
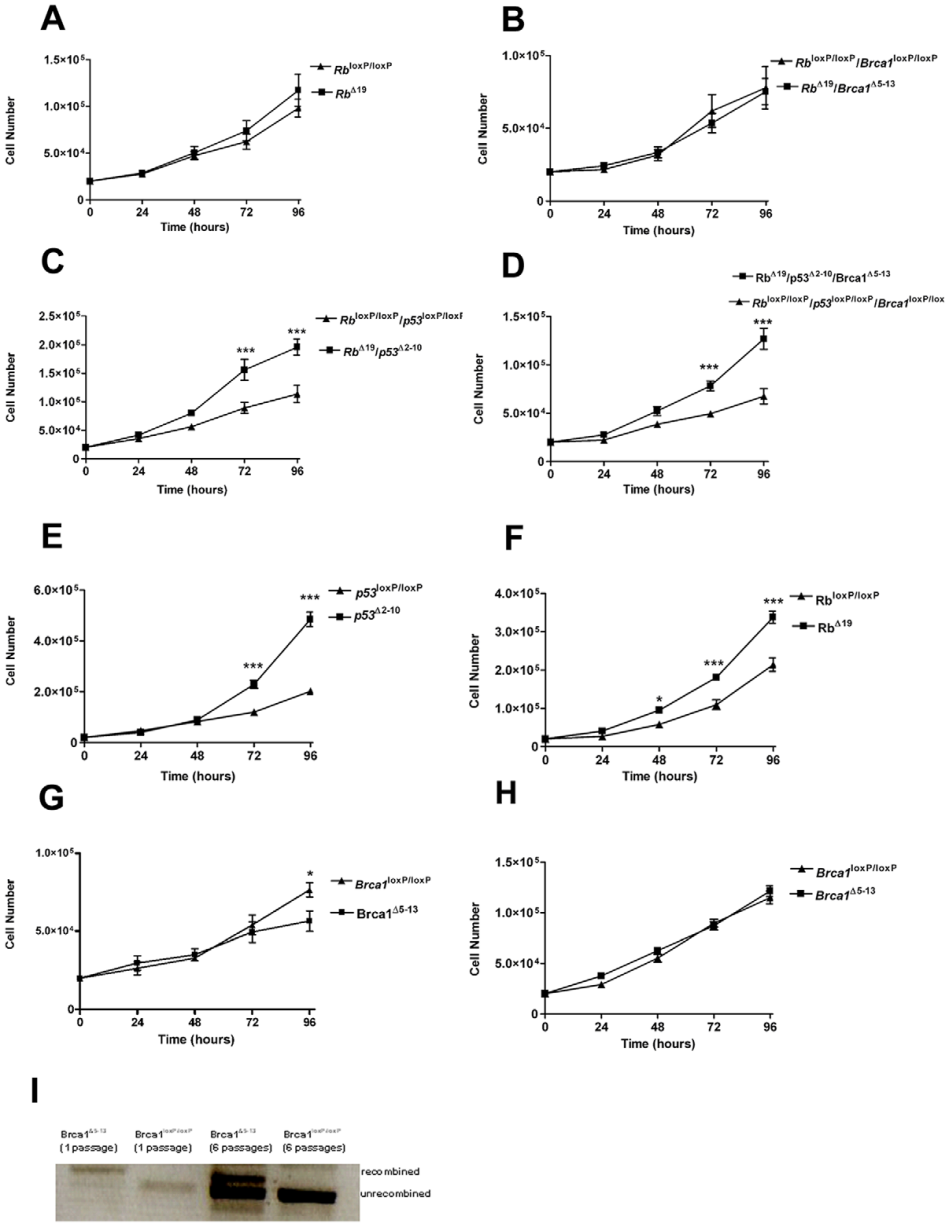
MOSE cell proliferation following inactivation of tumor suppressors. Number of viable MOSE cells evaluated 72 hours (A–D, G) or several passages (E–F, H) after infection with AdCre or AdGFP. **A**) *Rb* ^loxP/loxP^ MOSE cells evaluated 72 hours following infection with AdCre (Rb^Δ19)^ to inactivate *Rb* or with AdGFP (*Rb* ^loxP/loxP^). **B**) *Rb* ^loxP/loxP^/*Brca1* ^loxP/loxP^ MOSE cells infected with AdCre (Rb^Δ19^/*Brca1* ^Δ5-13^) to inactivate *Rb* and *Brca1* or with AdGFP (*Rb* ^loxP/loxP^/*Brca1* ^loxP/loxP^). **C**) *Rb* ^loxP/loxP^/*p53* ^loxP/loxP^ MOSE infected with AdCre (*Rb* ^Δ19^/*p53* ^Δ2-10^) to inactivate *Rb* and *p53* or AdGFP (*Rb* ^loxP/loxP^/*p53* ^loxP/loxP^). **D**) *Rb* ^loxP/loxP^/*p53* ^loxP/loxP^/*Brca1* ^loxP/loxP^ MOSE infected with AdCre (*Rb* ^Δ19^/*p53* ^Δ2-10^/*Brca1* ^Δ5-13^) or AdGFP (*Rb* ^loxP/loxP^/*p53* ^loxP/loxP^/*Brca1* ^loxP/loxP^). **E–F, H**) Proliferation of MOSE cells 4–9 passages following infection of AdCre or AdGFP. **E**) *p53* ^loxP/loxP^ vs. *p53* ^Δ2-10^ MOSE. **F**) *Rb* ^loxP/loxP^ vs. Rb^Δ19^ MOSE. **G**) *Brca1* ^loxP/loxP^ vs. *Brca1* ^Δ5-13^ MOSE evaluated 72 hours following infection with AdCre or AdGFP. **H**) *Brca1* ^loxP/loxP^ vs. *Brca1* ^Δ5-13^ MOSE, 4–9 passages following infection with AdCre or AdGFP. **I**) PCR of genomic DNA collected from cultured *Brca1* ^loxP/loxP^ OSE cells 1 or 6 passages following *in vitro* infection with AdCre (*Brca1* ^Δ5-13^) or AdGFP (*Brca1* ^loxP/loxP^). Error bars represent the SEM of data obtained from three experiments performed in triplicate. *, ** and *** indicate a significant difference where P<0.05, P<0.01 and P<0.001, respectively.

The appearance of non-epithelial tumors in the mice limited the investigation of the consequences of disruption of the tumor suppressor genes in OSE cells *in vivo*. We therefore isolated MOSE cells from mice with conditional expression of these genes and have previously demonstrated that inactivation of *Brca1* in MOSE cells resulted in decreased proliferation [36]. Inactivation of *p53* in *p53* ^loxP/loxP^ MOSE cells had no effect on proliferation, but proliferation was dramatically increased when both *Brca1* and *p53* were inactivated [36]. Here we show that, when growth rates were examined 72 hours after exposure to AdGFP or AdCre, conditional inactivation of *Rb* in *Rb* ^loxP/loxP^ MOSE cells had no effect on proliferation over the subsequent 96 hour period (**Figure 6A**). When both *Brca1* and *Rb* were inactivated in *Rb* ^loxP/loxP^/*Brca1* ^loxP/loxP^ MOSE cells, the proliferation defect seen when *Brca1* was inactivated alone (**Figure 6G**) was eliminated; however there was no significant increase in the rate of proliferation of the *Rb/Brca1*-deficient cells (**Figure 6B**). When *p53* was inactivated in conjunction with *Rb* in *Rb* ^loxP/loxP^/*p53* ^loxP/loxP^ MOSE cells, the resulting *Rb/p53*-deficient cells showed a dramatic increase in proliferation as compared to their control (AdGFP-infected) counterparts, resulting in double the number of cells counted after 72 and 96 hours (P<0.001; **Figure 6C**). When all three tumor suppressor genes were inactivated in the *Rb* ^loxP/loxP^/*p53^l^* ^oxP/loxP^/*Brca1* ^loxP/loxP^ MOSE cells, there was a significant increase in their proliferation compared to the AdGFP-infected cells (P<0.001; **Figure 6D**), resulting in a 2.7-fold increase in cell number after 96 hours.

When the proliferation rate of *p53* ^Δ2-10^ MOSE cells was examined several passages following AdCre infection, the *p53*-deficient MOSE had developed a significantly increased rate of proliferation compared to AdGFP-infected cells cultured for the same length of time (P<0.001, **Figure 6E**). After several passages, the *Rb* ^Δ19^ MOSE cells also had a significantly increased rate of proliferation, detectable as early as 48 hours (**Figure 6F**). In contrast, *Brca1*-deficient MOSE, which showed a reduced rate of proliferation immediately after inactivation of *Brca1* (**Figure 6G**), failed to show any difference from their AdGFP-infected counterparts after several passages (**Figure 6H**). While recombination at the loxP sites could still be detected in the AdCre-infected cells at this stage, non-recombined DNA was now predominant (**Figure 6I**), suggesting that the MOSE cells without recombination were outgrowing the slower growing *Brca1*-deficient cells.

### Effect of Inactivation of Multiple Tumor Suppressor Genes on Sensitivity to Cisplatin in MOSE Cells *In Vitro*

**Figure 7:**
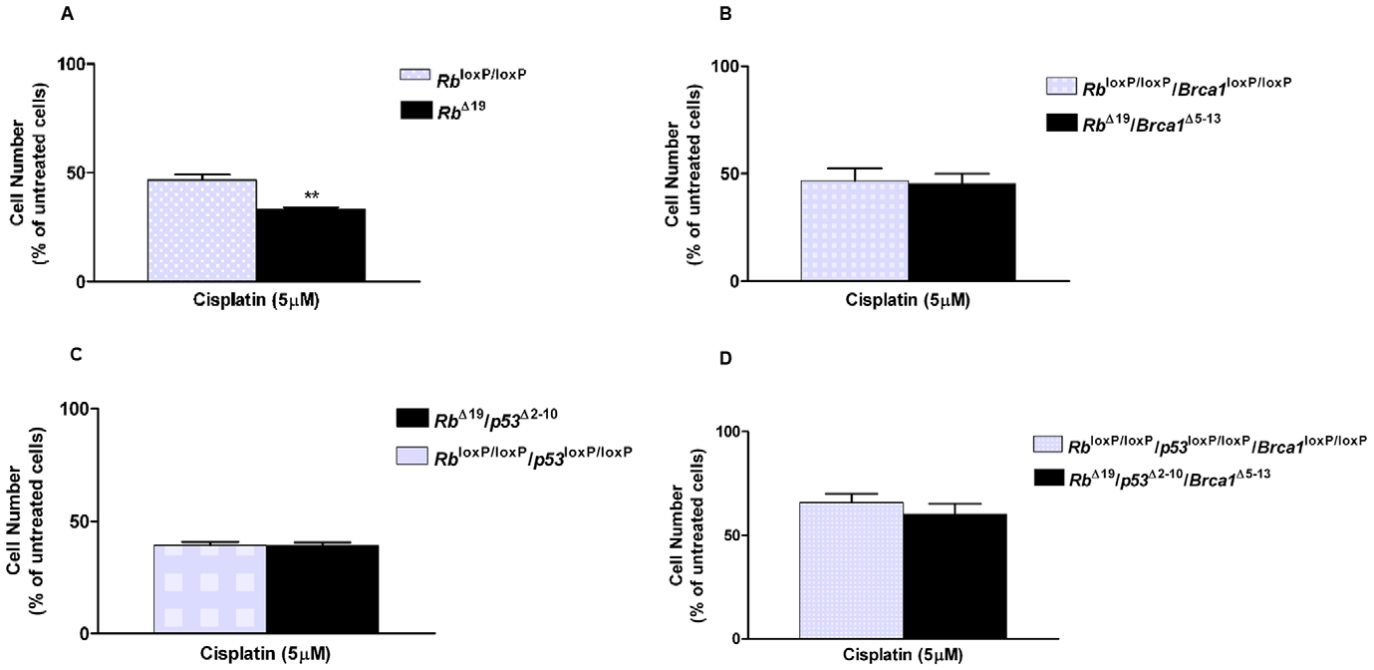
Treatment of MOSE cells with cisplatin following inactivation of multiple tumor suppressors. MOSE cells with specific inactivation of *Brca1*, *p53*, and/or *Rb* in various combinations were tested for their sensitivity to cisplatin by treatment with 5 µM cisplatin for 48 hours. **A**) *Rb* ^loxP/loxP^ MOSE cells infected with either AdCre (*Rb* ^Δ19^) or AdGFP (*Rb* ^loxP/loxP^); **B**) *Rb* ^loxP/loxP^/*Brca1* ^loxP/loxP^ MOSE cells infected with either AdCre (*Rb* ^Δ19^/*Brca1* ^Δ5-13^) or AdGFP (*Rb* ^loxP/loxP^/*Brca1* ^loxP/loxP^); **C**) *Rb* ^loxP/loxP^/*p53* ^loxP/loxP^ MOSE cells infected with either AdCre (*Rb* ^Δ19^/*p53* ^Δ2-10^) or AdGFP (*Rb* ^loxP/loxP^/*p53* ^loxP/loxP^); and **D**) *Rb* ^loxP/loxP^/*p53* ^loxP/loxP^/*Brca1* ^loxP/loxP^ MOSE cells infected with either AdCre (*Rb* ^Δ19^/*p53* ^Δ2-10^/*Brca1* ^Δ5-13^) or AdGFP (*Rb* ^loxP/loxP^/*p53* ^loxP/loxP^/*Brca1* ^loxP/loxP^). Values are the number of adherent cells presented as a proportion of similar cells exposed to the vehicle control (untreated cells). Error bars represent the SEM of three experiments performed in triplicate. **indicates a significant difference where P<0.01.

Inactivation of *Brca1* or *p53* alone in MOSE cells *in vitro* renders them significantly more sensitive to treatment with the chemotherapeutic agent cisplatin; however, when both *Brca1* and *p53* are conditionally inactivated, there is no significant difference between the sensitivities of *Brca1* ^Δ5-13^/*p53* ^Δ2-10^ and *Brca1* ^loxP/loxP^/*p53* ^loxP/loxP^ cells after 48 hours [36]. Here we show that the *Rb* ^Δ19^ MOSE cells were also significantly more sensitive to cisplatin treatment than the *Rb* ^loxP/loxP^ cells (33.28%±0.95% versus 46.85%±2.42%, P<0.01, viable cells expressed as a percentage of untreated cells, **Figure 7A**). However, when both *Rb* and *Brca1* or both *Rb* and *p53* were inactivated in MOSE cells, there was no difference in the proportion of cells remaining viable after 48 hours of treatment with cisplatin, relative to the AdGFP-infected controls (**Figure 7B,C**). The concomitant inactivation of all three tumor suppressor genes in the *Rb* ^loxP/loxP^/*p53* ^loxP/loxP^/*Brca1* ^loxP/loxP^ cells had no impact on the sensitivity of these cells to treatment with 5 µM of cisplatin for 48 hours compared to the corresponding control-infected cells (**Figure 7D**).

## Discussion

Our previous work showed that when *Brca1* was inactivated *in vivo* via intrabursal injection of AdCre, no tumorigenesis occurred when the animals were followed past one year post-injection, despite the development of significantly more premalignant changes in the OSE of these ovaries [36]. The frequent occurrence of *p53* mutations in ovarian cancers in women with *BRCA1* mutations [25], [26] and the apparent cooperation of Brca1 and p53 in mouse models of mammary cancers [27]–[31] led us to perform experiments in which conditional inactivation of *Brca1* was targeted to the OSE, along with the conditional inactivation the tumor suppressors *p53* and/or *Rb*. Our results show that invasive tumors developed in all mice that had inactivation of *p53* targeted to the OSE and that tumor development was accelerated in mice with concomitant inactivation of *Brca1*.

Based on the work of Flesken-Nikitin *et al.* [35], it was anticipated that the *Rb* ^Δ19^/*p53* ^Δ2-10^ mice would develop adenocarcinomas with little to no tumor formation seen when either *p53* or *Rb* was inactivated alone. In contrast to that study, the *Rb* ^Δ19^/*p53* ^Δ2-10^ mice in this study died of tumors that were classified as malignant leiomyosarcomas. Furthermore, conditional inactivation of *p53* alone led to the development of leiomyosarcomas in 100% of the mice. Ovarian sarcomas are rare in women [40], although aberrations in both the p53 and Rb pathways have been implicated in the development and progression of leiomyosarcomas [41]–[43], and mice that are null or heterozygous for *p53* are highly prone to developing lymphomas and sarcomas [44], [45]. Several groups have shown the capacity for OSE to develop into epithelial tumors using the method of intrabursal injection of AdCre for the activation or inactivation of genes [35], [36], [46], indicating that these cells are capable of transformation to epithelial cancers. The reasons for the prevalence of leiomyosarcomas are therefore unclear, but there are several possible factors that should be considered. First, the OSE is a poorly differentiated epithelium that is known to undergo epithelial-mesenchymal transition during the peri-ovulatory period [1], and it is possible that the inactivation of *p53* facilitated transformation more effectively in these mesenchymal cells. Second, although evidence from our lab and others [35], [36], [46] indicate that intrabursal delivery of adenoviruses results in infection of primarily surface epithelial cells, it may be possible that some underlying stromal cells are also infected and are more readily transformed than the epithelial cells.

A third reason for the occurrence of leiomyosarcomas in this study may be differences in the strain of mouse used. Genetic modifiers due to strain differences can influence the phenotype in mouse models of disease [47]–[49], so it is possible that strain differences may account for the discrepancies in both tumor incidence and histology. The animals utilized in the Flesken-Nikitin study [35] were backcrossed to the FVB/n strain, whereas the animals in this study were maintained on the FVB/n;129sv mixed strain background. Finally, it is possible that, in the strain used in this study, the smooth muscle cells of the bursal membrane, which would also be in contact with the adenoviral Cre recombinase, are more sensitive to the consequences of these recombination events than the OSE. The bursal membrane is largely composed of smooth muscle cells, much like the uterine and oviductal myometrium with which it is contiguous. Since the tumors that developed in these mice were generally found to be continuous with the bursal membrane, it may be that the tumors are arising from this tissue rather than the ovarian surface epithelium. We have recently found that Cre-mediated recombination can occur in cells in the bursal membrane and that cells carrying the recombined gene can still be found at least 65 days after intrabursal delivery of AdCre [50].

Despite this unanticipated phenotype, *Brca1*-deficiency in these tumors influenced the survival time of the mice, causing a significant reduction in the length of survival of *p53* and *p53/pRb*-deficient mice, compared to the mice without *Brca1* deficiency. Breast and ovarian cancers in women with *BRCA1* mutations arise at an earlier age than sporadic cancers [51], so it was interesting to observe that, despite the difference in histologic phenotype, inactivation of *Brca1* in mice resulted in tumors that developed more quickly. This action is in agreement with a recent report describing the ability of *BRCA1* deficiency to accelerate uterine leiomyosarcoma development [52]. The mechanism of this acceleration in tumor initiation and/or progression is unknown, although loss of Brca1 function is associated with genomic instability [19].

When MOSE cell proliferation was examined *in vitro*, the concomitant inactivation of *p53* rescued the growth suppression that was seen with inactivation of *Brca1* alone [36] and led to a substantially increased growth rate as compared to control cells. Loss of p53 function has been demonstrated to partially rescue the embryonic lethality of *Brca1* knockout mice and appears to play a critical role in mammary tumorigenesis in mice [27]–[31], [53]. Concomitant inactivation of both *p53* and *Rb* also resulted in a significant increase in proliferation in MOSE cells, an observation in agreement with results of similar studies reported previously [35]. Conditional inactivation of all three tumor suppressor genes, *Brca1*, *p53* and *Rb*, simultaneously in MOSE cells resulted in a dramatic increase in proliferation with significant increases in cell number with time over any of the other genotypic combinations. This increased rate of proliferation may explain the acceleration in tumor progression in cells lacking all three genes.

Inactivation of *p53* or *Rb* alone in MOSE cells had no immediate effect on proliferation, but after at least four passages, the growth characteristics became notably different, showing increased rates of proliferation relative to controls. These results suggest that, with prolonged culture, these cells may spontaneously acquire further genetic abnormalities that result in uncontrolled proliferation. The lack of any obvious morphological alterations in the OSE at 60 or 120 days after intrabursal AdCre injection in *p53/Rb*-deficient mice supports the hypothesis that tumor initiation requires additional genetic changes. The long latency in tumor development could be a result of the relatively low proliferative index of the OSE [54].

The increased sensitivity of MOSE to cisplatin treatment observed when only one tumor suppressor gene is inactivated appears to be lost when two or more are inactivated simultaneously. Cisplatin-resistant ovarian and cervical carcinoma cells have been shown to harbour multiple genetic changes, particularly affecting pathways leading to apoptosis that render the cells aberrantly tolerant of accumulated DNA damage [55], [56]. In this regard, familial ovarian cancers have a higher frequency of *p53* mutations than sporadic cancers [14], and it has been suggested that loss of p53 function is required for a cell to tolerate the loss of the Brca1 function [57]–[59]. Thus, loss of *Brca1* may result in compensation in the cell by the *p53* or *Rb* pathways, resulting in an increased apoptotic response to cisplatin. The concomitant loss of the *p53* or *Rb* expression may enable the cells to better tolerate cellular stresses, including exposure to cisplatin.

These results show similarities to mouse models of mammary tumorigenesis and uterine leiomyosarcomas in that *Brca1* inactivation can accelerate the initiation and/or progression of p53-mediated tumors. *Brca1* status, in combination with that of other tumor suppressor pathways, influence sensitivity of OSE cells to chemotherapeutic agents. The combination of tumor suppressors inactivated in this study failed to yield epithelial cancers, indicating that further analyses are needed to determine the mechanisms by which BRCA1 mutations cause transformation of the ovarian epithelium.

## References

[1] Auersperg N, Edelson MI, Mok SC, Johnson SW, Hamilton TC (1998). The biology of ovarian cancer.. Semin Oncol.

[2] Hirst JE, Gard GB, McIllroy K, Nevell D, Field M (2009). High rates of occult fallopian tube cancer diagnosed at prophylactic bilateral salpingo-oophorectomy.. Int J Gynecol Cancer.

[3] Shaw PA, Rouzbahman M, Pizer ES, Pintilie M, Begley H (2009). Candidate serous cancer precursors in fallopian tube epithelium of BRCA1/2 mutation carriers.. Mod Pathol.

[4] Levanon K, Crum C, Drapkin R (2008). New insights into the pathogenesis of serous ovarian cancer and its clinical impact.. J Clin Oncol.

[5] Roh MH, Kindelberger D, Crum CP (2009). Serous tubal intraepithelial carcinoma and the dominant ovarian mass: clues to serous tumor origin?. Am J Surg Pathol.

[6] Auersperg N, Wong AS, Choi KC, Kang SK, Leung PC (2001). Ovarian surface epithelium: biology, endocrinology, and pathology.. Endocr Rev.

[7] Sundfeldt K, Piontkewitz Y, Ivarsson K, Nilsson O, Hellberg P (1997). E-cadherin expression in human epithelial ovarian cancer and normal ovary.. Int J Cancer.

[8] Salazar H, Godwin AK, Daly MB, Laub PB, Hogan WM (1996). Microscopic benign and invasive malignant neoplasms and a cancer-prone phenotype in prophylactic oophorectomies.. J Natl Cancer Inst.

[9] Schlosshauer PW, Cohen CJ, Penault-Llorca F, Miranda CR, Bignon YJ (2003). Prophylactic oophorectomy: a morphologic and immunohistochemical study.. Cancer.

[10] Werness BA, Afify AM, Bielat KL, Eltabbakh GH, Piver MS (1999). Altered surface and cyst epithelium of ovaries removed prophylactically from women with a family history of ovarian cancer.. Hum Pathol.

[11] Finch A, Shaw P, Rosen B, Murphy J, Narod SA (2005). Clinical and pathologic findings of prophylactic salpingo-oophorectomies in 159 BRCA1 and BRCA2 carriers.. Gynecol Oncol.

[12] Berchuck A, Heron KA, Carney ME, Lancaster JM, Fraser EG (1998). Frequency of germline and somatic BRCA1 mutations in ovarian cancer.. Clin Cancer Res.

[13] Pal T, Permuth-Wey J, Betts JA, Krischer JP, Fiorica J (2005). BRCA1 and BRCA2 mutations account for a large proportion of ovarian carcinoma cases.. Cancer.

[14] Aunoble B, Sanches R, Didier E, Bignon YJ (2000). Major oncogenes and tumor suppressor genes involved in epithelial ovarian cancer (review).. Int J Oncol.

[15] King MC, Marks JH, Mandell JB (2003). Breast and ovarian cancer risks due to inherited mutations in BRCA1 and BRCA2.. Science.

[16] Geisler JP, Hatterman-Zogg MA, Rathe JA, Buller RE (2002). Frequency of BRCA1 dysfunction in ovarian cancer.. J Natl Cancer Inst.

[17] Russell PA, Pharoah PD, De FK, Ramus SJ, Symmonds I (2000). Frequent loss of BRCA1 mRNA and protein expression in sporadic ovarian cancers.. Int J Cancer.

[18] Chan KY, Ozcelik H, Cheung AN, Ngan HY, Khoo US (2002). Epigenetic factors controlling the BRCA1 and BRCA2 genes in sporadic ovarian cancer.. Cancer Res.

[19] Yu V (2000). Caretaker Brca1: keeping the genome in the straight and narrow.. Breast Cancer Res.

[20] Chen JJ, Silver D, Cantor S, Livingston DM, Scully R (1999). BRCA1, BRCA2, and Rad51 operate in a common DNA damage response pathway.. Cancer Res.

[21] Yoshida K, Miki Y (2004). Role of BRCA1 and BRCA2 as regulators of DNA repair, transcription, and cell cycle in response to DNA damage.. Cancer Sci.

[22] Larson JS, Tonkinson JL, Lai MT (1997). A BRCA1 mutant alters G2-M cell cycle control in human mammary epithelial cells.. Cancer Res.

[23] Yan Y, Spieker RS, Kim M, Stoeger SM, Cowan KH (2005). BRCA1-mediated G2/M cell cycle arrest requires ERK1/2 kinase activation.. Oncogene.

[24] Shao NS, Chai YL, Shyam E, Reddy P, Rao NV (1996). Induction of apoptosis by the tumor suppressor protein BRCA1.. Oncogene.

[25] Ramus SJ, Bobrow LG, Pharoah PD, Finnigan DS, Fishman A (1999). Increased frequency of TP53 mutations in BRCA1 and BRCA2 ovarian tumours.. Genes Chromosomes Cancer.

[26] Zweemer RP, Shaw PA, Verheijen RM, Ryan A, Berchuck A (1999). Accumulation of p53 protein is frequent in ovarian cancers associated with BRCA1 and BRCA2 germline mutations.. J Clin Pathol.

[27] Cressman VL, Backlund DC, Hicks EM, Gowen LC, Godfrey V (1999). Mammary tumor formation in p53- and BRCA1-deficient mice.. Cell Growth Differ.

[28] Deng CX (2001). Tumorigenesis as a consequence of genetic instability in Brca1 mutant mice.. Mutat Res.

[29] Liu X, Holstege H, van der Gulden H, Treur-Mulder M, Zevenhoven J (2007). Somatic loss of BRCA1 and p53 in mice induces mammary tumors with features of human BRCA1-mutated basal-like breast cancer.. Proc Natl Acad Sci U S A.

[30] Cressman VL, Backlund DC, Hicks EM, Gowen LC, Godfrey V (1999). Mammary tumor formation in p53- and BRCA1-deficient mice.. Cell Growth Differ.

[31] Xu X, Wagner KU, Larson D, Weaver Z, Li C (1999). Conditional mutation of Brca1 in mammary epithelial cells results in blunted ductal morphogenesis and tumour formation.. Nat Genet.

[32] Aprelikova ON, Fang BS, Meissner EG, Cotter S, Campbell M (1999). BRCA1-associated growth arrest is RB-dependent.. Proc Natl Acad Sci U S A.

[33] Wang A, Schneider-Broussard R, Kumar AP, MacLeod MC, Johnson DG (2000). Regulation of BRCA1 expression by the Rb-E2F pathway.. J Biol Chem.

[34] Fan S, Yuan R, Ma YX, Xiong J, Meng Q (2001). Disruption of BRCA1 LXCXE motif alters BRCA1 functional activity and regulation of RB family but not RB protein binding.. Oncogene.

[35] Flesken-Nikitin A, Choi KC, Eng JP, Shmidt EN, Nikitin AY (2003). Induction of carcinogenesis by concurrent inactivation of p53 and Rb1 in the mouse ovarian surface epithelium.. Cancer Res.

[36] Clark-Knowles KV, Garson K, Jonkers J, Vanderhyden BC (2007). Conditional inactivation of Brca1 in the mouse ovarian surface epithelium results in an increase in preneoplastic changes.. Exp Cell Res.

[37] Jonkers J, Meuwissen R, van der Gulden H, Peterse H, van der Valk M, Berns A (2001). Synergistic tumor suppressor activity of BRCA2 and p53 in a conditional mouse model for breast cancer.. Nat Genet.

[38] Vooijs M, van der Valk M, te Riele H, Berns A (1998). Flp-mediated tissue-specific inactivation of the retinoblastoma tumor suppressor gene in the mouse.. Oncogene.

[39] Sgagias MK, Wagner KU, Hamik B, Stoeger S, Spieker R (2004). Brca1-deficient murine mammary epithelial cells have increased sensitivity to CDDP and MMS.. Cell Cycle.

[40] Sood AK, Sorosky JI, Gelder MS, Buller RE, Anderson B (1998). Primary ovarian sarcoma: analysis of prognostic variables and the role of surgical cytoreduction.. Cancer.

[41] de Vos S, Wilczynski SP, Fleischhacker M, Koeffler P (1994). p53 alterations in uterine leiomyosarcomas versus leiomyomas.. Gynecol Oncol.

[42] Dei Tos AP, Maestro R, Doglioni C, Piccinin S, Libera DD (1996). Tumor suppressor genes and related molecules in leiomyosarcoma.. Am J Pathol.

[43] Leiser AL, Anderson SE, Nonaka D, Chuai S, Olshen AB (2006). Apoptotic and cell cycle regulatory markers in uterine leiomyosarcoma.. Gynecol Oncol.

[44] Donehower LA, Harvey M, Slagle BL, McArthur MJ, Montgomery CA (1992). Mice deficient for p53 are developmentally normal but susceptible to spontaneous tumours.. Nature.

[45] Jacks T, Remington L, Williams BO, Schmitt EM, Halachmi S (1994). Tumor spectrum analysis in p53-mutant mice.. Curr Biol.

[46] Dinulescu DM, Ince TA, Quade BJ, Shafer SA, Crowley D (2005). Role of K-ras and Pten in the development of mouse models of endometriosis and endometrioid ovarian cancer.. Nat Med.

[47] LeCouter JE, Kablar B, Hardy WR, Ying C, Megeney LA (1998). Strain-dependent myeloid hyperplasia, growth deficiency, and accelerated cell cycle in mice lacking the Rb-related p107 gene.. Mol Cell Biol.

[48] Simpson KJ, Wati MR, Deans AJ, Lindeman GJ, Brown MA (2004). MMTV-trBrca1 mice display strain-dependent abnormalities in vaginal development.. Int J Dev Biol.

[49] Woodworth CD, Michael E, Smith L, Vijayachandra K, Glick A (2004). Strain-dependent differences in malignant conversion of mouse skin tumors is an inherent property of the epidermal keratinocyte.. Carcinogenesis.

[50] Laviolette L, Garson K, Macdonald EA, Senterman MK, Courville K, Crane CA, Vanderhyden BC 17β-estradiol accelerates tumor onset and decreases survival in a transgenic mouse model of ovarian cancer.. Endocrinology 2009 (In press).

[51] Lynch HT, Casey MJ, Snyder CL, Bewtra C, Lynch JF (2009). Hereditary ovarian carcinoma: heterogeneity, molecular genetics, pathology, and management.. Mol Oncol.

[52] Xing D, Scangas G, Nitta M, He L, Xu X (2009). A role for BRCA1 in uterine leiomyosarcoma.. Cancer Res.

[53] Hakem R, de la Pompa JL, Elia A, Potter J, Mak TW (1997). Partial rescue of Brca1 (5–6) early embryonic lethality by p53 or p21 null mutation.. Nat Genet.

[54] Heller DS, Hameed M, Baergen R (2003). Lack of proliferative activity of surface epithelial inclusion cysts of the ovary.. Int J Gynecol Cancer.

[55] Lanzi C, Perego P, Supino R, Romanelli S, Pensa T (1998). Decreased drug accumulation and increased tolerance to DNA damage in tumor cells with a low level of cisplatin resistance.. Biochem Pharmacol.

[56] Perego P, Romanelli S, Carenini N, Magnani I, Leone R (1998). Ovarian cancer cisplatin-resistant cell lines: multiple changes including collateral sensitivity to Taxol.. Ann Oncol.

[57] Brugarolas J, Jacks T (1997). Double indemnity: p53, BRCA and cancer. p53 mutation partially rescues developmental arrest in Brca1 and Brca2 null mice, suggesting a role for familial breast cancer genes in DNA damage repair.. Nat Med.

[58] Cao L, Li W, Kim S, Brodie SG, Deng CX (2003). Senescence, aging, and malignant transformation mediated by p53 in mice lacking the Brca1 full-length isoform.. Genes Dev.

[59] Xu X, Qiao W, Linke SP, Cao L, Li WM (2001). Genetic interactions between tumor suppressors Brca1 and p53 in apoptosis, cell cycle and tumorigenesis.. Nat Genet.

